# Efficacy of Pre-Procedural Mouthwashes against SARS-CoV-2: A Systematic Review of Randomized Controlled Trials

**DOI:** 10.3390/jcm11061692

**Published:** 2022-03-18

**Authors:** Alvaro Garcia-Sanchez, Juan-Francisco Peña-Cardelles, Steve Ruiz, Flor Robles, Esther Ordonez-Fernandez, Angel-Orión Salgado-Peralvo, James Balloch, Jacob C. Simon

**Affiliations:** 1 Department of Oral Health and Diagnostic Sciences, School of Dental Medicine, University of Connecticut Health, Farmington, CT 06030, USA; balloch@uchc.edu (J.B.); jasimon@uchc.edu (J.C.S.); 2 Department of Health Sciences, Rey Juan Carlos University, 28040 Madrid, Spain; 3 Oral and Maxillofacial Surgery Department, School of Dental Medicine, University of Connecticut Health, Farmington, CT 06030, USA; 4 Department of Prosthodontics, School of Dental Medicine, University of Connecticut Health, Farmington, CT 06030, USA; 5 Division of General Dentistry, School of Dental Medicine, University of Connecticut Health, Farmington, CT 06030, USA; sruiz@uchc.edu (S.R.); roblesmijangos@uchc.edu (F.R.); eordonezfernandez@uchc.edu (E.O.-F.); 6 Department of Stomatology, Faculty of Dentistry, University of Seville, 41009 Seville, Spain; orionsalgado@hotmail.com

**Keywords:** COVID-19, SARS-CoV-2, mouthwashes, aerosols, chlorhexidine, povidone-iodine, cetylpiridinium chloride, hydrogen peroxide, colony-forming units

## Abstract

The oral mucosa is one of the first sites to be affected by the SARS-CoV-2. For this reason, healthcare providers performing aerosol-generating procedures (AGPs) in the oral cavity are at high risk of infection with COVID-19. The aim of this systematic review is to verify whether there is evidence in the literature describing a decrease in the salivary viral load of SARS-CoV-2 after using different mouthwashes. An electronic search of the MEDLINE database (via PubMed), Web of Science, SCOPUS, and the Cochrane library database was carried out. The criteria used were those described by the PRISMA^®^ Statement. Randomized controlled trial studies that have used mouthwashes as a form of intervention to reduce the viral load in saliva were included. The risk of bias was analyzed using the Joanna Briggs Institute Critical Appraisal Tool. Ultimately, eight articles were included that met the established criteria. Based on the evidence currently available in the literature, PVP-I, CHX and CPC present significant virucidal activity against SARS-CoV-2 in saliva and could be used as pre-procedural mouthwashes to reduce the risk of cross-infection.

## 1. Introduction

Severe acute respiratory syndrome coronavirus 2 (SARS-CoV-2) or COVID-19 was first discovered in December 2019 in Wuhan (China) followed by a rapid worldwide spread in a short duration of time [1]. This disease presented as an atypical pneumonia with the potential involvement of multiple body systems and organs [2]. As of 14 January 2022, there has been a total of 320,488,206 confirmed cases and 5,538,159 deaths worldwide [3].

Evidence shows that SARS-CoV-2 can be transmitted by direct contact, droplets and fomites, and through airborne transmission [4,5,6]. Droplets of saliva have an important role in the transmission of the virus between people. These droplets are usually generated during activities, such as speech or coughing, and when inhaled, ingested or in direct contact with the mucosa, infection occurs [7,8].

The oral mucosa acts as a portal of entry by the virus [6]. SARS-CoV-2 can invade oral and salivary gland epithelium due to the great amounts of angiotensin converting enzyme 2 (ACE2) receptors in these locations [9,10,11]. The interaction between the spike protein of the virus and the ACE2 receptors allows for the entry of the virus into cells [12]. This emphasizes the role of the oral cavity in the transmission of the virus.

For this reason, healthcare providers performing aerosol-generating procedures (AGPs) in the oral cavity are at high risk of infection with COVID-19 [13]. Most dental treatments are AGPs due to the use of ultrasonic devices, high-speed handpieces and 3-in-1 air-water syringes, among others. There are also numerous AGPs performed routinely in hospitals where airway manipulation is required. Since the onset of the pandemic, personal protective equipment (PPE) has been one of the most important measures to prevent transmission in healthcare facilities. In addition, pre-procedural rinses or mouthwashes have been proposed to reduce the viral load in saliva and to reduce the number of colony-forming units (CFUs) in aerosols [14,15]. Moreover, various organizations recommend their use before dental treatments [16,17,18].

The available literature includes various systematic reviews evaluating in vitro and in vivo studies. In the last few months, a significant number of randomized control trials (RCTs) investigating the efficacy of different mouthwashes against SARS-CoV-2 have published their results.

Therefore, the aim of this systematic review of RCTs was to evaluate the efficacy of different mouthwashes on the reduction of salivary viral load of SARS-CoV-2 in COVID-19 positive patients confirmed with RT-PCR tests as pre-procedural rinses.

## 2. Materials and Methods

### 2.1. Protocol

The present review was performed according to the Preferred Reporting Items for Systematic Reviews and Meta-Analyses (PRISMA^®^) Statement [19,20]. The protocol was registered at the International Prospective Register of Systematic Reviews (PROSPERO) under the registration number CRD42022303574.

### 2.2. Focused Question

This investigation was designed to answer the following PICO (P = patient/problem/population; I = intervention; C = comparison; O = outcome) question based on the PRISMA^®^ guidelines.

In patients diagnosed with COVID-19 (P), does the use of pre-procedural mouthwashes (I) compared to not prescribing them (C) reduce the viral load present in saliva (O)?

### 2.3. Eligibility Criteria

Prior to the search, inclusion and exclusion criteria were defined.

#### 2.3.1. Inclusion Criteria

Type of studies: (a) RCTs (b) studies conducted in humans in which participants had a reverse-transcription polymerase chain reaction (RT-PCR) examination positive for SARS-CoV-2; (c) studies published in English.

#### 2.3.2. Exclusion Criteria

Excluded studies include the following: (a) animal studies; (b) experimental laboratory studies; (c) studies whose study base focused on other areas besides the oral cavity saliva and/or oropharynx; (d) studies that did not evaluate the reduction of viral load in saliva; (e) non-randomized controlled trials; (f) systematic reviews and meta-analyses; (g) literature review studies; (h) case reports; (i) letters to the editor; (j) abstracts or conference papers; (k) comments; and (l) unpublished articles.

### 2.4. Information Sources and Search Strategy

The search was conducted in four different electronic databases: MEDLINE (via PubMed), SCOPUS, the Cochrane Library database and the Web of Science (WoS).

The search strategy was carried out by two authors independently (A.G.-S. and A.-O.S.-P.). There were no time restrictions and it was updated to January 2022. MeSH (Medical Subjects Headings) terms, keywords and other free terms were used with Boolean operators (OR, AND) to combine searches: (‘mouthwash’ OR ‘oral rinse’ OR ‘mouth rinse’ OR ‘povidone iodine’ OR ‘chlorhexidine chloride’ OR ‘hydrogen peroxide’ OR ‘cetylpyridinium chloride’ OR ‘essential oil’ OR ‘phthalocyanine derivatives’ OR ‘ethanol’ OR ‘citrox’ OR ‘listerine’) AND (‘COVID-19’ OR ‘SARS-CoV-2’ OR ‘SARS’). The search in different platforms followed the syntax rules of each database. We also screened articles present in the reference lists of the included articles.

### 2.5. Study Records

The results were independently compared by two authors (A.G.-S. and A.-O.S.-P.) to guarantee completeness and removal of duplicates by a reference manager software. Next, the title and abstract of the remaining articles were reviewed individually. Ultimately, full-text papers were selected following the criteria previously described. Disagreements over eligible articles were resolved including a third author (J.-F.P.-C.), to reach a consensus. Data collection of the included studies was performed using an excel spreadsheet.

### 2.6. Risk of Bias Assessment

The methodology of eligible studies was evaluated following the Joanna Briggs Institute (JBI) Critical Appraisal Tool [21] by two independent authors (A.G.-S. and J.-F.P.-C.). The studies were categorized as low-quality (0–7 domains) or high-quality assessment (8–13 domains). A third author (A.-O.S.-P.) was included to resolve any disagreements between the two authors.

## 3. Results

### 3.1. Study Selection

**Figure 1:**
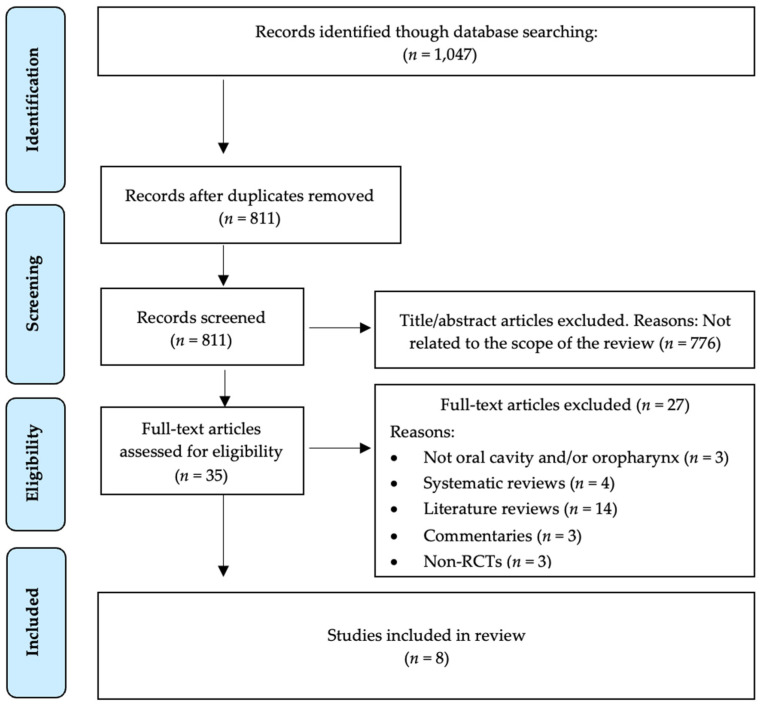
PRISMA^®^ flow diagram of the search processes and results.

The search strategy resulted in 1047 articles. There were 236 duplicates, therefore, 811 remained. Then, two authors (A.G.-S. and A.-O.S.-P.) independently examined the titles and abstracts and excluded 776 that were beyond the scope of this study. Consequently, we obtained 35 possible references. After full text review of the 35 papers, 27 were excluded because they investigated areas other than oral cavity saliva and/or oropharyngeal saliva (*n* = 3), were systematic reviews (*n* = 4), literature reviews (*n* = 14), commentaries (*n* = 3) and non-RCTs (*n* = 3). Therefore, eight studies were included in our systematic review (Figure 1).

### 3.2. Study Characteristics

All the studies included were RCTs published in 2020 and 2021. There was a large discrepancy among the sample sizes of selected articles (ranged from 36 to 294). Due to the low number of studies available, it was decided that there would be no exclusion criteria set for a minimum number of participants. The total number of patients included within the studies was 851. All these patients were RT-PCR positive for SARS-CoV-2.

In these studies, rinsing times ranged between 30 s and 1 min. In the placebo group, distilled water [14,22,23,24,25], saline [26], and an unspecified inactive substance [27] were used. The control group in one study did not receive treatment [28]. In the test group several active compounds were used: CDCM^®^ (0.1% beta-cyclodextrin and 0.1% Citrox^®^) (*n* = 1 [14]); hydrogen peroxide (HP) at 1% [25,26] or 1.50% [22]; chlorhexidine (CHX) at 0.12% [22,25,26,27,28] or 0.20% [23,24]; a combination of 0.12% CHX and 1.50% HP [22]; cetylpyridinium chloride (CPC) 0.07% [25] and 0.075% [23]; a combination of 0.075% CPC and 0.28% Zinc (Zn) [22]; and povidone iodine (PVP-I) at 0.50% [23,26], 1% [24] and 2% [25]. All the studies investigated the effect of oral rinses, except for one article [28] which studied the combination of an oral rinse with an oropharyngeal spray. All saliva samples were evaluated through RT-PCR. Baseline samples were collected immediately before the intervention. The number of saliva samples after interventions varied among the studies. Two studies collected one sample of saliva after intervention [24,28], three collected two samples [22,26,27], and three collected three samples [14,23,25]. A summary of the findings of the included articles are described in Table 1.

The main findings of the articles are described below.

Carrouel et al. [14] (2021) investigated the effects of a commercially available mouthwash containing CDCM^®^ on the reduction of the salivary viral load of SARS-CoV-2 versus (vs.) a placebo group (distilled water). A total of 176 patients with a confirmed diagnosis of COVID-19 were randomly assigned to the intervention or the control group. The patients rinsed three times a day for 7 days. Saliva sampling was performed before the first mouthwash and 1 h before the remaining two rinses. Saliva was then tested using RT-PCR. The use of this compound had a significant effect on reducing viral load 4 h after the initial dose, but the reduction was moderate at 7 days.

Eduardo et al. [22] (2021) recruited 60 patients and randomly allocated them into 5 groups: Placebo (distilled water), 0.075% CPC + 0.28% Zinc, 1.5% HP, 0.12% CHX and 1.50% HP + 0.12% CHX. Saliva samples for RT-PCR were collected at baseline, 30 and 60 min after rinse. CPC + Zinc and CHX resulted in significant reductions of the SARS-CoV-2 viral load in saliva up to 60 min after rinsing, while HP mouthwash resulted in a significant reduction up to 30 min after rinsing.

Chaudhary et al. [26] (2021) performed a randomized, triple-blinded study evaluating the effect of normal saline (placebo), 1% HP, 0.12% CHX and 0.50% PVP-I in 40 patients. Saliva samples for RT-PCR were collected at 15 and 45 min after rinsing. All 4 mouth rinses, including the placebo group, decreased viral load by 61–89% at 15 min and by 70–97% at 45 min.

Seneviratne et al. [23] (2020) evaluated the reduction in viral load using 0.50% PVP-I, 0.20% CHX, 0.075% CPC and placebo (distilled water) as mouthwashes. Saliva samples were taken for RT-PCR at baseline (pre-rinse), 5 min, 3 h and 6 h post-rinsing. Comparison of salivary Cycle threshold (Ct) values within all groups at 5 min, 3 h and 6 h did not show any significant differences. Compared with the control group (distilled water), a significant decrease in the viral load in the CPC group at 5 min and 6 h and in the PVP-I group at 6 h was observed.

Elzein et al. [24] (2021) recruited 61 patients with a confirmed diagnosis of COVID-19 who were randomly allocated into three groups: 1% PVP-I and 0.20% CHX as test groups and distilled water as the control group. Saliva samples were taken at baseline and 5 min post-rinse. A significant difference was noted between the delta Ct of distilled water wash and each of the test groups (0.20% CHX and 1% PVP-I). On the other hand, no significant difference was found between test groups.

Huang et al. [28] (2021) investigated 0.12% CHX as a mouthwash and as an oropharyngeal spray to reduce the viral load in saliva of SARS-CoV-2. Two hundred and ninety-four patients were recruited and randomly allocated into four groups: oral rinse only, oral rinse combined with oropharyngeal spray, and two control groups. The two control groups did not receive treatment. Oropharyngeal swabs were collected 4 days post-rinse for RT-PCR. SARS-CoV-2 was eliminated from the oropharynx in 62.1% of patients who used CHX as an oral rinse, vs. 5.50% of the control group. In the combination group, 86% eliminated oropharyngeal SARS-CoV-2, vs. 6.30% of control patients.

Ferrer et al. [25] (2021) investigated the reduction of the salivary load of SARS-CoV-2 in a sample of 80 patients randomly allocated into 5 groups: 2% PVP-I, 1% HP, 0.07% CPC, 0.12% CHX and control (distilled water). Saliva samples were taken at baseline, 30 min, 60 min and 120 min after the mouth rinse, and were evaluated through RT-PCR. There were no statistically significant changes in virucidal activity after the use of different mouthwashes compared with the control group.

Costa et al. [27] (2021) recruited 100 patients for a randomized double-blind control trial evaluating 0.12% CHX as a mouthwash vs. a control group (an inactive substance). Saliva samples were taken at baseline, 5 min and 60 min after rinsing, and were evaluated through RT-PCR. There was a significant reduction in the salivary viral load at both 5 min and 60 min after rinsing compared with the control group. Total reductions in the salivary viral load were 72% in the CHX group vs. 30% in the control group.

### 3.3. Risk Bias Assessment

Using the JBI Critical Appraisal Tool for RCTs [21], we established that one paper [28] had a low-quality assessment (0–7 domains) and seven papers [14,22,23,24,25,26,27] had a high-quality assessment (8–13 domains). The analysis of the articles included is shown in Table 2.

## 4. Discussion

SARS-CoV-2 is easily transmitted via droplets of saliva by direct contact with the ocular, nasal or oral mucosa, as well as through airborne transmission when AGPs are performed in closed spaces. Not only the dental practitioners are affected, but those health professionals involved in bronchoscopies, endoscopies, or intubations, among others, are at higher risk of infection [29]. Rigorous infection control methods are essential in clinical settings, and pre-procedural mouthwashes could potentially be advantageous to decrease the risk of cross-infection between patients and personnel.

CHX has been widely proven as an effective antiplaque and antigingivitis solution [30,31]. In vitro studies demonstrated that it has virucidal activity against HSV-1 and Influenza A [32]. In addition, it can reduce bacterial levels in aerosol [15]. The concentrations of CHX varied between 0.12% [22,25,26,27,28] and 0.20% [23,24] among the included studies. CHX was the most predominant mouthwash present with seven out of the nine studies including it as one of their intervention groups. The study by Costa et al. [27] found that there was a significant reduction (72%) in the salivary load at both 5 min and 60 min after rinsing compared with the control when 0.12% CHX was used. Three of the included studies demonstrated a significantly reduced viral load using 0.12% [22,26] and 0.20% [28] concentrations compared with the control group. These findings are consistent with in vitro studies where the virucidal activity seen was >99.99% at a concentration of 0.20% and 99.99% at a concentration of 0.12% at both 30 s and 60 s contact times [33]. Xu et al. [34] had similar results, but the contact time was 30 min. On the other hand, the study by Ferrer et al. [25] found no statistically significant changes in viral load after the use of 0.12% CHX. Similar results have been obtained with in vitro studies, where the reduction of the viral load was not significant using 0.10% and 0.20% CHX [35,36,37].

PVP-I is composed of iodine and the water-soluble polymer polyvinylpyrrolidone. It has bactericidal and virucidal activity against MERS-CoV and influenza A, among others [38,39]. The common use of PVP-I as a mouthwash has no deleterious health effects [40]. However, its use is contraindicated in patients allergic to iodine, thyroid disease and pregnancy [41]. During the initial stages of the pandemic, several associations recommended the use of pre-procedural 0.20% PVP-I mouth rinse to decrease the risk of SARS-CoV-2 transmission [16,17,18]. The concentrations of PVP-I in the included studies varied. Out of the 5 included studies that used PVP-I as a solution, two of them used 0.50% [24,26], one used 1% [28] and the last used 2% [25]. In general, the included studies demonstrate the efficacy of PVP-I to reduce the salivary load of SARS-CoV-2 [26,28].

Seneviratne et al. [23] showed virucidal activity at 5 min and 3 h of post-rinsing with 0.5% PVP-I, but only a statistically significant increase at 6 h post-rinsing. Similar results have been reported in the literature. Multiple studies demonstrated virucidal activities of >4 log 10 at 15 [42], 30 [43,44] and 60 s [33,45] of contact time. In contrast, the study by Ferrer et al. [25] showed no statistically significant changes in the viral load when using 2% PVP-I as a rinse.

HP is a widely used antimicrobial agent and it is effective against several viruses including adenovirus, rhinovirus, myxovirus and influenza A [46]. Three studies included evaluated this solution at concentrations of 1% [25,26] and 1.50% [22]. One of the included studies found that rinsing with HP resulted in a significant reduction of salivary viral load up to 30 min after rinsing, but the reduction at 60 min was not significant [22]. Similarly, the study by Chaudhary et al. [26] showed that the use of 1% HP mouthwash resulted in significant reductions of 80–89%, comparable to the other mouthwashes evaluated in the study (CHX, PVP-I). On the other hand, the study by Ferrer et al. [25] did not find statistically significant changes in salivary viral load after the use of the 1.50% HP mouthwash. An in vitro study reported log reductions of <1 at 30 s contact time using 1.50% HP. This reduction was significantly lower than all other mouthwashes studied (PVP-I, CHX, Ethanol + essential oils) [36]. Furthermore, a prospective clinical pilot study found that 1% HP does not reduce the intraoral viral load in SARS-CoV-2 positive patients [47].

CPC is a well-known, broad-spectrum quaternary ammonium used in over-the-counter mouthwashes with a rapid bactericidal effect on gram-positive pathogens and a fungicidal effect on yeasts in particular [48,49]. The concentrations remained constant (0.075%) in the three articles included [22,23,25]. Seneviratne et al. [23] showed a significant reduction in viral load with the use of CPC mouthwash at 6 h, comparable with the reduction seen with PVP-I. Similarly, Eduardo et al. [22] found a significant reduction in viral load for up to 60 min after rinsing when using a combination of CPC + Zinc. Comparable results have been described in the literature. A study by Rodríguez-Casanovas et al. [50] evaluated a commercial mouthwash containing a combination of 0.05% CPC and 0.20% D-limonene. They observed a significant reduction of about 6 logs in the viral load compared with the control.

CDCM^®^ is a commercially available mouthwash composed of Beta-cyclodextrin and Citrox^®^. There was only one study included in this systematic review evaluating this compound [14]. They showed that the use of CDCM^®^ had a significant effect on reducing viral load 4 h after the initial dose but the reduction was moderate at 7 days. No other studies regarding the efficacy of this compound against SARS-CoV-2 have been published. However, a study on the efficacy of Citrox^®^ on reducing the oral microbiota concluded that Bioflavonoid preparations of Citrox^®^ have a broad spectrum of antimicrobial activity against oral microorganisms [51].

There are other solutions with a potential virucidal effect on SARS-CoV-2 in saliva. Two in vitro studies evaluated the efficacy of commercially available products containing essential oils (i.e., Listerine^®^) [34,36]. They significantly reduced the viral titer (≥3.11 log 10), and they were as effective as PVP-I. Iota-Carrageenan (IC) is a derivative from red marine algae with virucidal activity that has been demonstrated in vitro against rhinovirus, herpesviruses and influenza A [52,53]. An in vitro study by Bansal et al. [54] found that inhibitory concentrations are easily achievable by nasal and nebulization formulations which significantly reduced the viral load when compared with untreated controls. Nevertheless, further clinical studies are needed to demonstrate their efficacy and safety.

### 4.1. Strengths and Limitations

This systematic review presents several strengths, including an unrestricted search in the literature, data retrieval and risk assessment performed in duplicate.

However, this review also presents some limitations. There was a large discrepancy in the number of participants in the studies included. Additionally, concentrations of the mouthwashes were not homogeneous in different studies. COVID-19 is a disease that is continuously being investigated, and multiple RCTs are evaluating the use of different mouthwashes in progress at this moment. Our results must be interpreted with caution until further investigations are carried out.

### 4.2. Recommendations for Further Research

This study systematically reviewed the first RCTs performed on this topic. Further in vitro studies evaluating potential new molecules and additional RCTs are essential to demonstrate the safety and effectiveness of different mouthwashes.

## 5. Conclusions

Within the limitations of this study, PVP-I, CHX and CPC present significant virucidal activity against SARS-CoV-2 in saliva. A 30 s pre-procedural rinse of 0.50/1% PVP-I, 0.12/0.20% CHX, or 0.075% CPC could be beneficial to reduce the risk of cross-infection in healthcare settings performing AGPs.

## Figures and Tables

**Table 1:** Results of the included RCTs.

Author/ Year	Sample Size	Time of Testing	Intervention/Duration of Rinses		Conclusions
			Control Group	Test Group(s)	
Carrouel et al. [14] (2021)	176	Rinsed 3 times daily. Saliva collected at baseline, 1 h ^1^ before the two following rinses and last taken 1 h after the 2nd rinse.	Placebo (distilled water)/ 1 min ^2^	30 mL ^3^ of 0.1% beta-cyclodextrin and 0.1% Citrox^®^ rinse (CDCM^®^)/ 1 min.	CDCM^®^ was effective at 4 h post-rinse. At day 7, only a modest virucidal activity was observed.
Eduardo et al. [22] (2021)	60	Saliva samples for RT-PCR ^4^ collected at baseline, 30 and 60 min after rinse.	Placebo (distilled water)/ 1 min.	0.075% CPC ^5^ + 0.28% Zn ^6^ (30 s ^7^), 1.5% HP ^8^ (1 min), 0.12% CHX ^9^ (30 s) or 1.5%HP + 0.12% CHX (1 min + 30 s).	CPC + Zinc and CHX were effective in reducing the salivary viral load 60 min post-rinse. HP significantly reduced only at 30 min post-rinse.
Chaudhary et al. [26] (2021)	40	Two samples of saliva taken at 15 and 45 min post-rinse.	Placebo (normal saline), 1%/ 60 s.	1% HP, 0.12% CHX, 0.5% PVP-I ^10^. Rinsed with 15 mL/ Intervals of 30 s each for 60 s total.	All 4 mouthwashes reduced the salivary load by 61–89% at 15 min and by 70–97% at 45 min.
Elzein et al. [24] (2021)	61	Saliva was collected at baseline and 5 min after rinsing.	Placebo (distilled water)/ 30 s.	1% PVP-I and 0.2% CHX/ 30 s.	The Ct ^11^ of the intervention groups (CHX 0.20% and 1% PVP-I) was significantly different compared to the control group.
Huang et al. [28] (2021)	294	Oropharyngeal swab collected 4 days post-rinse for RT-PCR.	Untreated control group.	0.12% CHX/ 30 s 2/day and 0.12% CHX/30 s 2/day + oropharyngeal spray (1.5 mL) 3 times daily.	SARS-CoV-2 was eliminated from the oropharynx in 62.1% of patients who used CHX as an oral rinse, vs. 5.5% of the control group. In the combination group, 86.0% eliminated oropharyngeal SARS-CoV-2, vs. 6.3% of control patients.
Ferrer et al. [25] (2021)	84	RT-PCR at baseline, 30, 60 and 120 min after mouthrinse	Placebo (distilled water)/ 1 min.	2% PVP-I, 1% HP, 0.07% CPC, 0.12% CHX/ 1 min	None of the mouthwashes evaluated presented a statistically significant change in the salivary viral load.
Costa et al. [27] (2021)	100	RT-PCR at baseline, 5 and 60 min after rinsing	Placebo (inactive substance).	15 mL of 0.12% CHX/ 1 min.	There was a significant reduction in the salivary load at both 5 and 60 min after rinsing compared with the control. There was a reduction in the load of SARS-CoV-2 in 72% of the volunteers using chlorhexidine vs. 30% in the control group.
Seneviratne et al. [23] (2020)	36	Saliva samples for RT-PCR taken at baseline, 5 min, 3 and 6 h after rinse.	Placebo (water)/ 30 s.	0.5% PVP-I, 0.2% CHX, 0.075% CPC/30 s.	There were no differences in the reduction of salivary load in all intervention groups. When compared with the control group, PVP-I and CPC showed a significant reduction at 6 h. CPC also showed a significant reduction at 5 min.

^1^ h., hour(s); ^2^ min., minutes; ^3^ mL., milliliters; ^4^ RT-PCR., reverse-transcription polymerase chain reaction; ^5^ CPC., cetylpyridinium chloride; ^6^ Zn., zinc; ^7^ s., seconds; ^8^ HP., hydrogen Peroxide; ^9^ CHX., chlorhexidine; ^10^ PVP-I., povidone iodine; ^11^ Ct, cycle threshold.

**Table 2:** JBI Critical Appraisal Tool [21] for RCTs. Reprinted with permission from JBI. Copyright 2020.

Critical Appraisal Questions	Carrouel et al. [14] (2021)	Eduardo et al. [22] (2021)	Chaudhary et al. [26] (2021)	Seneviratne et al. [23] (2020)	Elzein et al. [24] (2021)	Huang et al. [28] (2021)	Ferrer et al. [25] (2021)	Costa et al. [27] (2021)
1. Was true randomization used for assignment of participants to treatment groups?	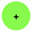	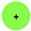	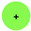	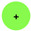	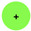	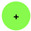	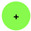	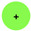
2. Was allocation to treatment groups concealed	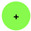	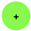	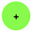	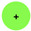	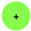	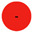	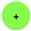	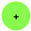
3. Were treatment groups similar at the baseline?	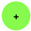	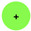	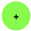	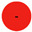	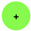	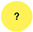	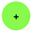	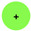
4. Were participants blind to treatment assignment?	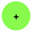	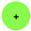	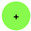	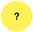	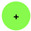	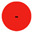	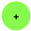	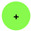
5. Were those delivering treatment blind to treatment assignment?	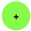	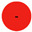	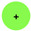	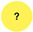	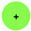	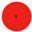	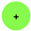	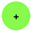
6. Were outcome assessors blind to treatment assignment?	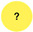	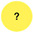	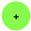	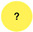	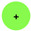	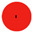	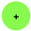	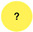
7. Were treatment groups treated identically other than the intervention of interest?	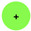	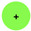	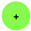	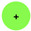	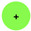	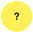	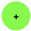	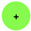
8. Was follow up complete and if not, were differences between groups in terms of their follow-up adequately described and analyzed?	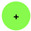	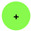	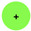	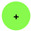	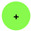	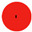	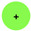	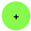
9. Were participants analyzed in the groups to which they were randomized?	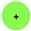	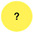	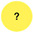	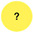	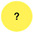	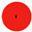	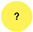	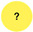
10. Were outcomes measured in the same way for treatment groups?	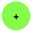	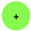	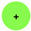	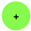	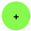	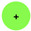	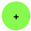	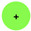
11. Were outcomes measured in a reliable way?	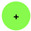	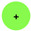	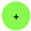	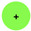	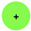	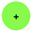	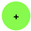	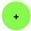
12. Was appropriate statistical analysis used?	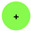	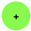	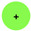	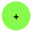	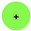	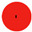	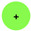	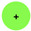
13. Was the trial design appropriate and any deviations from the standard RCT design accounted for in the conduct and analysis of the trial?	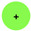	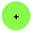	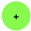	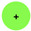	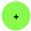	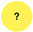	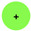	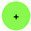

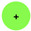
= yes, 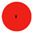
= no, 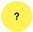
= uncertain.

## Data Availability

Data available in a publicly accessible repository.
